# Inhibition of Microbial Quorum Sensing Mediated Virulence Factors by *Pestalotiopsis sydowiana*

**DOI:** 10.4014/jmb.1907.07030

**Published:** 2020-01-23

**Authors:** Paramanantham Parasuraman, B Devadatha, V. Venkateswara Sarma, Sampathkumar Ranganathan, Dinakara Rao Ampasala, Dhanasekhar Reddy, Ranjith Kumavath, In-Won Kim, Sanjay K. S. Patel, Vipin Chandra Kalia, Jung-Kul Lee, Busi Siddhardha

**Affiliations:** 1 Department of Microbiology, School of Life Sciences, Pondicherry University, Puducherry 605014, India; 2 Department of Biotechnology, School of Life Sciences, Pondicherry University, Puducherry 605014, India; 3 Centre for Bioinformatics, School of Life Sciences, Pondicherry University, Puducherry 605014, India; 4 Department of Chemical Engineering, Konkuk University, Seoul 05029, Republic of Korea; 5 Department of Genomic Science, School of Biological Sciences, Central University of Kerala, Tejaswini Hills, Periya (P.O), Kasaragod, Kerala 671320, India

**Keywords:** *Pestalotiopsis sydowiana*, *Pseudomonas aeruginosa*, anti-biofilm, anti-quorum sensing, gene expression, in silico

## Abstract

Quorum sensing (QS)-mediated infections cause severe diseases in human beings. The control of infectious diseases by inhibiting QS using antipathogenic drugs is a promising approach as antibiotics are proving inefficient in treating these diseases. Marine fungal (*Pestalotiopsis sydowiana* PPR) extract was found to possess effective antipathogenic characteristics. The minimum inhibitory concentration (MIC) of the fungal extract against test pathogen *Pseudomonas aeruginosa* PAO1 was 1,000 μg/ml. Sub-MIC concentrations (250 and 500 μg/ml) of fungal extract reduced QS-regulated virulence phenotypes such as the production of pyocyanin, chitinase, protease, elastase, and staphylolytic activity in *P. aeruginosa* PAO1 by 84.15%, 73.15%, 67.37%, 62.37%, and 33.65%, respectively. Moreover, it also reduced the production of exopolysaccharides (74.99%), rhamnolipids (68.01%), and alginate (54.98%), and inhibited the biofilm formation of the bacteria by 90.54%. In silico analysis revealed that the metabolite of *P. sydowiana* PPR binds to the bacterial QS receptor proteins (LasR and RhlR) similar to their respective natural signaling molecules. Cyclo(-Leu-Pro) (CLP) and 4-Hydroxyphenylacetamide (4-HPA) were identified as potent bioactive compounds among the metabolites of *P. sydowiana* PPR using in silico approaches. The MIC values of CLP and 4-HPA against *P. aeruginosa* PAO1 were determined as 250 and 125 μg/ml, respectively. All the antivirulence assays were conducted at sub-MIC concentrations of CLP (125 μg/ml) and 4-HPA (62.5 μg/ml), which resulted in marked reduction in all the investigated virulence factors. This was further supported by gene expression studies. The findings suggest that the metabolites of *P. sydowiana* PPR can be employed as promising QS inhibitors that target pathogenic bacteria.

## Introduction

Quorum sensing (QS) regulates the infectious diseases in bacteria [1]. It operates through the signaling molecules, that is, autoinducers. The QS system in bacteria coordinates and influences the expression of genes responsible for the secretion of various virulence factors and biofilm formation, which lead to pathogenicity. Inhibition of QS-mediated gene expression can help control bacterial infection and biofilm development without affecting their growth pattern [2, 3]. Due to this unique feature, the bacteria develop less resistance to QSIs compared to antibiotics [4]. In recent years, various QS inhibitors of chemical and biological origin have been reported [5-8].

*Pseudomonas aeruginosa* is an opportunistic human pathogen that causes several clinical complications including chronic lung infection in cystic fibrosis patients [9, 10]. *P. aeruginosa* infection operates via the QS-mediated expression of several virulence traits like elastase, lipopolysaccharide, rhamnolipids, pyocyanin, cyanide, and exotoxin as well as flagellar motility, biofilm maturation, antimicrobial resistance, and alginates, which lead to biofilm formation [11]. Rhamnolipids play a significant role in evading the host immune response and facilitate the bacteria in successfully establishing the infection [12]. The infection system of *P. aeruginosa* is coordinated by acyl-homoserine lactone (AHL) genes for the two QS systems, that is, LasIR and RhlIR. Both of these systems get activated in a cascade manner by their specific signaling molecules, 3-oxo-C_12_-HSL and C4-HSL, respectively [9, 13]. Drugs are being developed to effectively target the QS mechanism in order to control the *P. aeruginosa* infection [14].

Bioactive compounds from diverse organisms are gaining immense attention as QSIs [1, 15-24]. The present study aims to discover novel QSIs from the marine fungus against the biofilm-forming ability of *P. aeruginosa* PAO1, an opportunistic pathogen.

## Materials and Methods

### Microbial Strains, Culture Media, and Conditions

Fungi from dry wood samples were isolated on an agar plate and grown in 2% malt extract medium at 28 ± 2°C for 21 days. For the extraction of crude metabolites, the fungal isolates were cultivated in malt extract broth at same incubation conditions as aforementioned. *P. aeruginosa* PAO1, gifted by Dr. E. Peter Greenberg (Department of Microbiology, University of Washington and School of Medicine, USA), was cultured on Luria-Bertani (LB) agar plate or broth at 37°C. *Chromobacterium violaceum,* biosensor strain was cultured at 37°C in LB medium. For bioassay on plate to evaluate the effect of fungal crude extract on the production of violacein, an active culture of *C. violaceum* was inoculated in LB soft agar (0.8% agar) at 1.5 × 10^8^ CFU/ml cell density and this mixture was plated on the surface of LB agar plate.

### Collection of Samples, Isolation of Fungi, and Preparation of Crude Extract

Different dry wood samples were collected from the coast near the village of Muthupet, Cuddalore District, Tamil Nadu, India. Isolation of fungal species was executed by applying single spore isolation technique on malt extract agar. Morphologically distinct colonies of fungi were isolated and stored at −80°C with 25% glycerol. The selected fungal isolates were cultured for crude extract. Briefly, the fungi were subcultured in malt extract broth for 24 days and incubated at static conditions at 28 ± 2°C by adding 2% of inoculum in the culture medium. The cell-free supernatant of the culture broth was collected after centrifugation at 10,000 ×*g* for 15 min. To this, ethyl acetate (1:1 v/v) was mixed and stirred well for 1 h. The organic phase was isolated and dried using the rotary evaporator [25]. The dried crude extracts of the fungi were diluted in 10% sterile DMSO to prepare the stock solution of 2 mg/ml and were used in the screening assays against the indicator strain. The process of fungal crude extraction was performed in three independent experiments and studied for anti-QS activity. All the fungal isolation and crude extraction procedures in this study were performed in the Fungal Toxicology Lab, Department of Biotechnology, Pondicherry University.

### Screening of Fungal Isolates for Their Violacein Inhibition Activity

Inhibition of violacein production in *C. violaceum* by fungal isolates was determined using the double layer agar diffusion method. Briefly, on a solid LB agar plate, the molten LB soft agar was seeded with 1.5 × 10^8^ CFU/ml of indicator strain and allowed to solidify. Wells were prepared using 8 mm metal borer and filled with two different concentrations of fungal crude extract and incubated at 37°C for 24 h. Inhibition of violacein production was represented as zone of inhibition in mm [26].

### Identification of Fungal Isolates

The fungal isolate presenting good anti-QS activity was identified by amplifying ITS region using ITS1 (5’ TCCGTAGGTGAACCTGCGG 3’) as forward primer and ITS4 (5’ TCCTCCGCTTATTGATATGC 3’) as reverse primer (Macrogen Inc., Seoul, South Korea). The nucleotide sequence result was equated with the DNA sequence in NCBI GenBank (https://blast.ncbi.nlm.nih.gov/Blast.cgi) to determine the closely related species. The ITS gene sequence of fungal isolate (PPR) was deposited to the NCBI GenBank database. Phylogeny was inferred using the neighbor-joining method in MEGA 5.04 software after aligning the sequences with CLUSTAL_W, and bootstrap analysis was carried out with 1000 replications [27].

### Determination of Minimum Inhibitory Concentration (MIC) and Growth Curve Analysis

MIC of the fungal crude extract was determined using the microdilution method in order to study the effect of subinhibitory concentration (Sub-MIC) on QS-regulated virulence factors of the test bacterium, *P. aeruginosa* [28]. About 2 mg/ml of fungal crude extract was used as the initial concentration and was diluted 2-fold in LB broth to a total volume of 200 μl in the microtiter plates. The wells were inoculated with 2 μl of bacterial suspension (1.5 × 10^8^). The assay was performed in triplicate and the sample was incubated at 37°C for 24 h. After incubation, the absorbance of each well was recorded at 600 nm.

Growth curve analysis was performed to determine the effect of sub-MIC of fungal crude extract on the growth of the test pathogen, *P. aeruginosa* PAO1 [29]. Overnight bacterial suspension was inoculated into 0.1 L of LB broth in 0.25 L conical flask, and the fungal extracts (250 and 500 μg/ml) were added. The flasks were incubated at 37°C on a rotary incubator at 120 rpm and the absorbance of broth was monitored at 600 nm for every 1 h up to 24 h.

### Violacein Inhibition Assay

The potential of the fungal extract in suppressing the violacein synthesis in *C. violaceum* was quantified by employing the procedure followed by Ma *et al.* [30]. The overnight grown *C. violaceum* was altered to an optical density of 0.5 at 600 nm. Aliquots of 100 μl were inoculated in the test tube containing 2 ml of LB and incubated with or without adding the sub-MIC concentrations (250 and 500 μg/ml) of fungal extract. The tubes were incubated at 37°C for 18 h. The culture suspensions were centrifuged at 10,000 ×*g* for 15 min and the bacterial pellet was collected. Next, 1 ml of DMSO was added to every tube and vigorously shaken for 1 h. The bacterial culture treated with 10% DMSO serving as a negative control. The suspensions were centrifuged at 12,000 ×*g* for 10 min, the supernatants were collected, and the optical density of the supernatant was determined at 590 nm.

### Pyocyanin Production

Quantitative determination of the effect of fungal extract on pyocyanin synthesis in *P. aeruginosa* was investigated, as described previously, with minor modifications [31]. The supernatant of overnight grown *P. aeruginosa* PAO1, in the presence or absence of fungal extract, was collected and used to extract the pyocyanin pigment from the culture supernatant. The optical density of the resultant pink-colored solution was recorded at 520 nm.

### Azocasein-Degrading Proteolytic Activity

The effect of fungal extract on protease synthesis in *P. aeruginosa* was determined by azocasein assay. The cell-free supernatant of the overnight cultured *P. aeruginosa* PAO1 in presence or absence of fungal extract was imparted to azocasein (0.5% in 100 mM Tris buffer with pH 8), followed by incubation at 28°C for 1 h. After the incubation, 10% trichloroacetic acid was added to the reaction mixture and incubated at 4°C for 20 min. The mixture was then centrifuged at 12,000 ×*g* for 10 min and supernatant was collected. Equal volumes of 1 M NaOH solution were added to the supernatant and the absorbance was measured at 420 nm [26].

### Elastase and Chitinase Assay

Elastolytic activity was determined by Elastin-Congo red (ECR) method [32]. Supernatant (0.1 ml) obtained from the overnight grown *P. aeruginosa,* with or without treatment of fungal extract, was added to 0.9 ml of ECR buffer (0.1 M Tris, 0.01 M CaCl_2_, pH 7.5) and incubated at 37°C for 4 h on a rotary shaker. The reaction was hindered by adding 100 μl of 0.12 M EDTA at 4°C. The reaction mixture was centrifuged (10,000 ×*g* for 15 min) to remove the insoluble ECR, and the absorbance of the supernatant was recorded at 495 nm.

Chitinase assay was performed by adopting the method described previously with slight modifications [33]. The culture supernatant obtained from the overnight grown *P. aeruginosa* PAO1, with or without treatment with sub-MIC concentrations of fungal extract, was added in 2:1 with 100 mM sodium citrate buffer (pH 4.8), also containing chitin azure (0.05%). The reaction mixture was incubated on a rotary shaker at 37°C for a week. After the incubation period, the absorbance of the reaction mixture was recorded spectrophotometrically at 570 nm.

### Staphylolytic Activity

Staphylolytic assay was performed to determine the effect of fungal extract on the LasA protease behavior of supernatant obtained for *P. aeruginosa* PAO1 on boiled *Staphylococcus aureus* cells [34]. The cell-free supernatant (100 μl) was added to 0.9 ml of the overnight cultured *P. aeruginosa* PAO1 in presence or absence of the fungal extract of boiled *S. aureus* suspension. Supernatant of the *P. aeruginosa* PAO1, cultured with 10% DMSO, was considered as a negative control. The change in the optical density was measured at 0- and 60-min. Activity was expressed as the percentage inhibition compared to control.

### Swimming and Swarming Motility Assay

Swimming and swarming motility was determined as described elsewhere [35]. Briefly, on the medium (1% tryptone, 0.5% NaCl, and 0.3% agar) for swimming and for swarming (1% peptone, 0.5% NaCl, 0.5% D-fructose, and 0.6% agar) amended with or without the fungal extract, was point inoculated with the test bacterium and incubated at 37°C for 24 h. After the incubation period, the plates were compared with the control (treated with 10% DMSO).

### Effect of Fungal Extract Against the Biofilm of *P. aeruginosa* PAO1

The effect of fungal extract on biofilm development in *P. aeruginosa* was evaluated by a method described by Yin *et al.* [36]. The test bacterium was cultured in LB broth supplemented with 1% glucose in presence or absence of fungal extract, in a microtiter plate at 37°C for 24 h. After incubation, the culture suspension was separated and washed gently to remove the planktonic cells in the wells. The biofilm was stained with 0.1% crystal violet solution and the excess stain was removed using repeated washings. The dye adsorbed to the biofilm was solubilized using ethanol and measured spectrophotometrically at 570 nm.

### Exopolysaccharide (EPS) Production

EPS production by the test bacterium was qualitatively determined using Congo red agar (CRA) plate method [37]. The test bacterium, *P. aeruginosa,* was cultured on the CRA medium (3.7% brain-heart infusion broth, 3.6% sucrose, 1.5% agar, and 0.08% Congo red), with or without fungal extract, and was incubated at 37°C for 24 h. Supernatant obtained from the culture broth was mixed with chilled absolute ethanol and left overnight at 4°C to precipitate EPS. The amount of the EPS was estimated by employing the method of Husain *et al.* [33], and the result was represented as percentage inhibition.

### Rhamnolipid Quantification

The effect of fungal extract on rhamnolipid production by *P. aeruginosa* was evaluated using the Orcinol method, as explained by Luo *et al.* [38]. One-milliliter supernatant of *P. aeruginosa* PAO1, cultured with or without fungal extract, was altered to pH 2.0, with HCl. An equal volume of ethyl acetate was added to the supernatant to solubilize the metabolites and the organic phase was separated and completely dried. The dried compounds were dissolved in 0.1 ml of milliQ water and 0.9 ml of orcinol solution [0.19% orcinol solution was prepared in 53% (v/v) H_2_SO_4_]. The mixture was incubated at 80°C for 30 min and the optical density was measured at 421 nm.

### Alginate Production

The effect of fungal extract on synthesis of alginate by *P. aeruginosa* PAO1 was investigated using the method described by Gopu *et al.* [39]. Briefly, the 70 μl of overnight cultured *P. aeruginosa* PAO1, in the presence or absence of the fungal extract, was mixed with 600 μl of boric acid–sulphuric acid mixture at a ratio of 4:1 under cold conditions. The mixture was vortexed and 20 μl of 0.2% carbazole dissolved in ethanol was added. The reaction mixture was vortexed for 10 sec and incubated at 55°C for 30 min. The optical density of the reaction mixture after incubation was measured at 530 nm.

### Microscopic Analysis

The effect of fungal extract on the biofilm development in *P. aeruginosa* PAO1 was determined via microscopic analysis as described previously by Kalia *et al.* [29]. The test bacterium was cultured on a cover slip, in presence or absence of fungal extract, and was washed with phosphate-buffered saline (PBS) to remove the unadhered cells. For light microscopic observation, the cover slips were stained with 0.4% crystal violet solution; whereas, for confocal laser scanning microscopy (CLSM), the biofilm-coated cover slips were stained with 0.01% acridine orange solution and incubated for 10 min. After incubation, the excess stain was removed by washing several times with sterile PBS and visualized under a light microscope (100× magnification) and CLSM (40× magnification).

### Gas Chromatography-Mass Spectrometry (GC-MS) Analysis

The metabolites present in the crude extract were determined using GC-MS analysis. The GC model Clarus 680 with an ion-trap mass spectrometer model Clarus 600 (EI), equipped with Elite-5MS column having specifications of 30 m in length and 0.25 mm in thickness of the film, was employed for this experiment. The compounds were detected using an electron ionization system, which uses higher energy electrons (70 eV). Helium gas was applied as a carrier gas with a constant run of 1 ml/min. The temperature at the initial stage was set as 260°C and programmed with an increasing rate of 10°C per min during the chromatographic run. Next, 1 μl of fungal extract diluted in ethyl acetate was injected. By using GC-MS NIST (2008), the bioactive compounds present in the fungal extract were identified [40].

### Receptor Protein Retrieval, Modeling, and Validation

The ligand binding domain of LasR was retrieved from the Protein Data Bank (PDB) ID:2UV0. The three-dimensional (3D) structure of the RhlR receptor molecule was built from the protein sequence retrieved from UniPort database (ID: P54292.1) in the ROBETTA server for protein structure modeling. The overall stereo chemistry quality assessment of the generated model of the RhlR structure was validated in the RAMPAGE web server (http://mordred.bioc.cam.ac.uk/~rapper/rampage.php) [41]. The best RhlR 3D-model after validation was selected for the docking studies.

### Molecular Docking

The docking studies were carried out in Schrodinger Maestro software version 11.5 (Schrodinger, LLC, New York, NY, 2018) in order to understand the interaction of fungal metabolites against two major QS receptor proteins, LasR and RhlR, using the natural ligands, 3-oxo-C_12_-HSL and C_4_-HSL as reference molecules, respectively. The LasR and RhlR ligand binding domain molecule protein preparation was carried out using the protein preparation wizard of Maestro suite with default settings. The grid files were generated individually using Glide, version 7.8, in the Maestro software. For LasR, the docking site was generated around its autoinducer (3-oxo-C_12_-HSL) interacting residues, and the grid for RhlR was defined vicinity of the active site residue Trp-68 of its natural autoinducer (C_4_-HSL) [42]. The known anti-QS molecules biacelein and furanone C30 were used as positive controls for the QS receptors LasR and RhlR, respectively. The 3D structure files of all fungal metabolites from GC-MS analysis were collected from the PubChem database and prepared in the LigPrep module of Maestro software version 11.5 with enabled Epik option where other settings were set as default. The prepared receptor grids and ligands were docked in Glide, version 7.8 in Maestro suite, with enabled Extra Precision mode [43]. LigPlot+ version 1.4.5 and Chimera version 1.6.2 were used for 2D and 3D analysis, respectively. Among the identified compounds, two compounds, cyclo(-Leu-Pro) (CLP) and 4-hydroxyphenyl acetamide (4-HPA), showed promising docking scores with the QS receptors similar to those of the natural ligands. Thus, these compounds were subjected to the molecular dynamics simulation analysis to investigate the conformational changes in the QS receptor proteins following interaction with the natural ligands or bioactive compounds.

### Molecular Dynamics Simulation

The results of molecular docking did not reveal the conformational changes found globally among the receptor proteins after interaction with their respective natural ligands or bioactive compounds, since the docking scores of interaction between the receptor and the ligand were determined at a particular region at a specific time. Through the molecular dynamics simulation study, we were able to detect the unwanted modification in the topology of the protein. The complexes of QS receptor proteins with selected bioactive compounds were simulated using GROMOS force field in the GROMACS v5.1.2 software [44]. The system was equilibrated under NVT and NPT ensembles before a final MD run of 50,000 ps with a time step of 2 fs. Post-simulation binding free energy of the complex formed by QS receptor protein and its natural ligand or bioactive compound and the interaction energy values were estimated using the Molecular Mechanics Poisson-Boltzmann Surface Area (MM/PBSA) analysis [45].

### Effect of Bioactive Compounds of *P. sydowiana* PPR Crude Extract on *P. aeruginosa* PAO1

Based on the in silico results, the bioactive compounds CLP and 4-HPA were observed to show promising anti-QS properties, which were further confirmed by evaluating the bioactive compounds for their anti-QS activity against *P. aeruginosa* PAO1 [46]. The commercially available CLP (Spec-Chem, China) was dissolved in methanol (2 mg/ml), whereas 4-HPA (Alfa Aesar, India) was dissolved in sterile distilled water (2 mg/ml). Baicalein (Sigma-Aldrich, India), used as a positive control in this study, was dissolved in methanol (2 mg/ml). Varying concentrations of these commercially available bioactive compounds were prepared (800, 400, 200, 100, 50, 25, and 12.5 μg/ml) in respective test tubes containing 1 ml of Mueller Hinton broth and 1% of *P. aeruginosa* PAO1 followed by incubation at 37°C for 18 h. The cell density of *P. aeruginosa* PAO1 treated with or without the bioactive compounds was measured at 600 nm using UV-Visible spectrophotometer, and MIC was calculated.

The sub-MIC values of CLP and 4-HPA (100 and 62.5 μg/ml) were tested for anti-QS potential by assessing pyocyanin, chitinase, elastase, and staphylolytic activities as well as motility inhibition and reduction in biofilm formation according to the protocols mentioned above.

### Gene Expression Studies

The effect of fungal bioactive compounds on the virulence genes of *P. aeruginosa* PAO1 was tested using RT-PCR. The test pathogen was grown in the LB broth with or without bioactive compounds at 37°C for 24 h. After incubation, the bacterial cell pellets were harvested by centrifugation at 10,000 ×*g* for 5 min. The cell pellets were resuspended in TRIzol reagent (Sigma-Aldrich, India) and total RNA was isolated according to the manufacturer’s guidelines. The purity and concentration of the RNA were measured using Nanodrop (Thermo Fisher Scientific, USA) and the isolated RNA was preserved at –80°C. The RNA was converted into cDNA using a RevertAid First Strand cDNA synthesis kit (Thermo Scientific, USA) according to the manufacturer’s instructions.

RT-PCR was performed in a total volume of 10 μl containing SYBR Green Master Mix (Thermo Scientific, USA) on a Roche Light Cycle 480 system using appropriate primers (Table S1). The PCR cycling conditions were as follows: initial denaturation at 95°C for 10 min followed by denaturation at 95°C for 30 sec, annealing for 15 sec as mentioned in Table S1 and extension at 72°C for 15 min. These conditions were maintained for 45 cycles and the final extension was performed at 72°C for 5 min [46]. All samples were analyzed in triplicates and normalized to *pr°* gene expression.

### Statistical Analysis

All the experiments were conducted thrice, and the data of the assays were expressed as mean values with standard deviation.

## Results

### Isolation and Screening of the Anti-QS Potential of Fungi

Fungi with anti-QS potential were isolated from the dry wood samples collected from the marine environment. A total of 14 distinct colonies of fungi were obtained as pure cultures using the single-spore isolation technique. The crude extracts from 14 fungal isolates showed variable degree of inhibition on violacein and pyocyanin production by *C. violaceum* and *P. aeruginosa*, respectively (Table 1). The halo inhibition zones around the wells were observed. Among the 14 fungal isolates, PPR revealed promising violacein inhibition activity without exhibiting bactericidal activity. The fungal isolate PPR was selected further for batch fermentation process, followed by crude extract preparation and determination of anti-QS activity.

### Characterization of Fungal Isolate

**Fig. 1:**
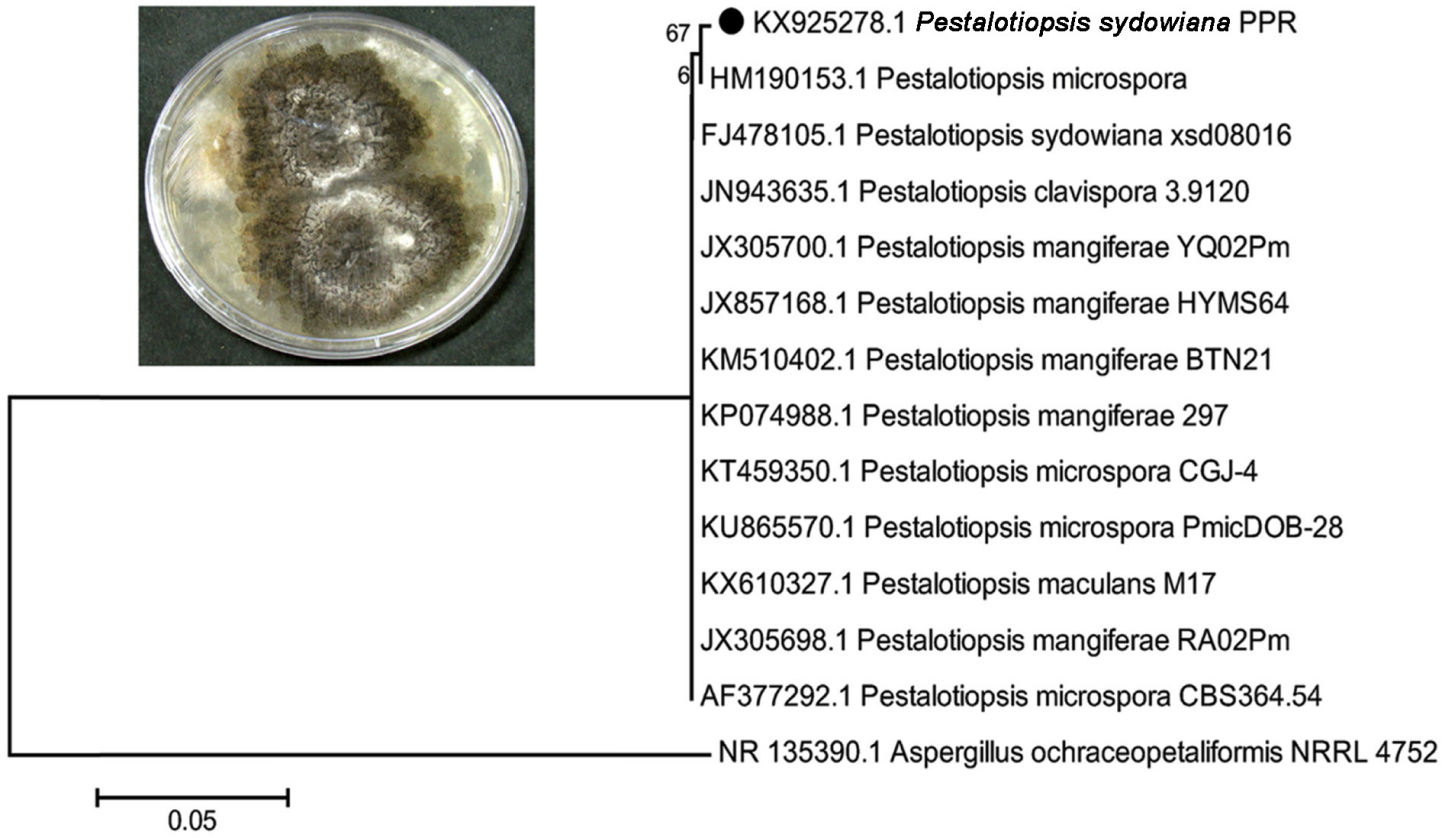
Culture plate image of *Pestalotiopsis sydowiana* PPR and phylogenetic tree based on ITS rRNA sequences using neighbor-joining of the strain *P. sydowiana* PPR. Branch distances represent the nucleotide substitution rate and scale bar represents the changes per nucleotide position.

The light and phase contrast microscopic observations of PPR isolate presented an appendage-bearing conidial anamorphic form. The partial ITS rRNA gene sequence revealed 99% similarity with *Pestalotiopsis sydowiana.* The resulted sequence was aligned with closely associated *Pestalotiopsis* sp. obtained from the NCBI GenBank database. The resulted phylogenetic tree revealed similarity with *P. sydowiana* with a resemblance matrix bootstrap value of 67 (Fig. 1). The GenBank accession number for the obtained partial ITS rRNA gene sequence of *P. sydowiana* strain is KX925278.

### Effect of Crude Extract on *P. aeruginosa* PAO1 Growth

**Fig. 2:**
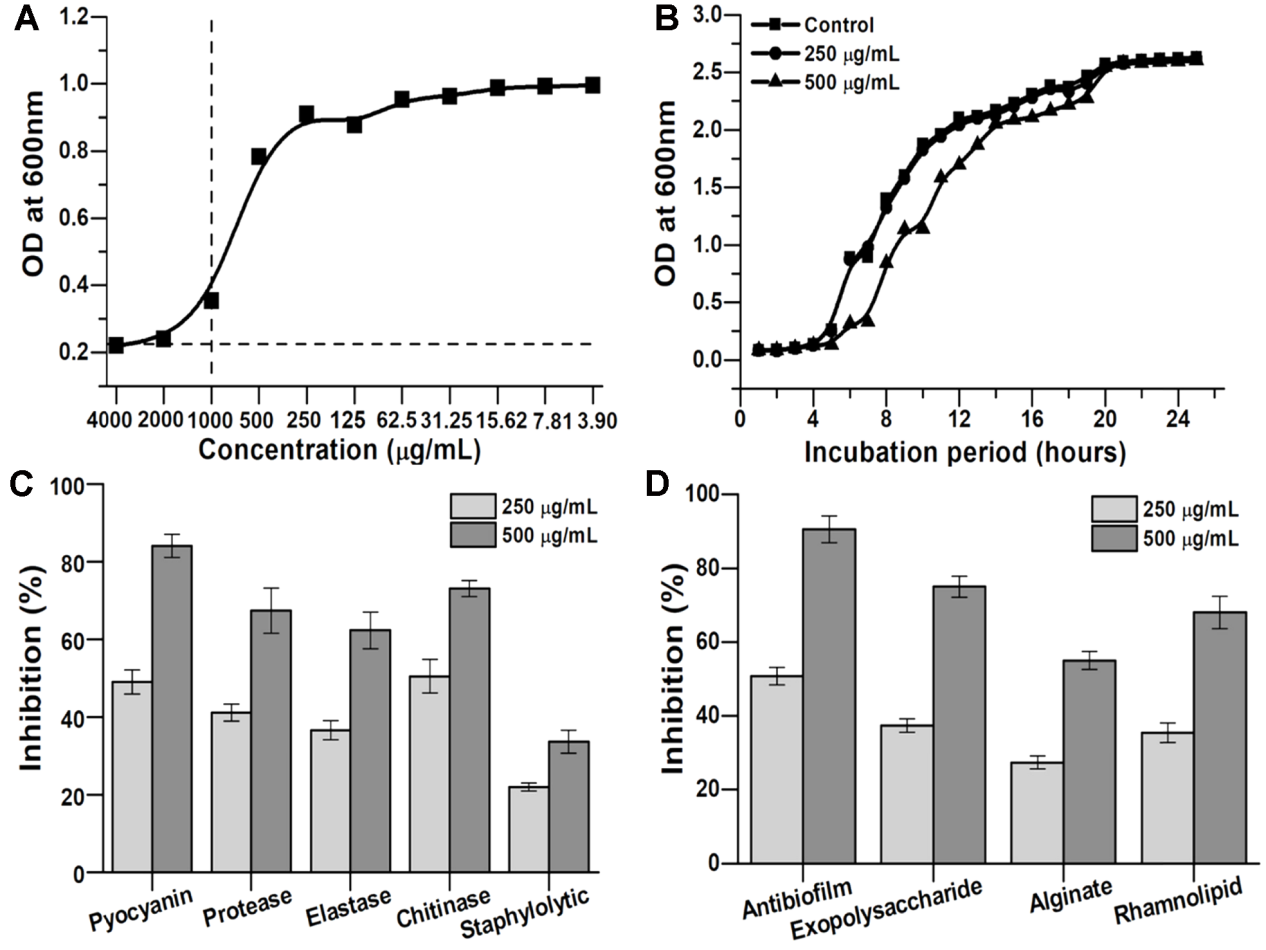
(A) Minimum inhibitory concentration of *Pestalotiopsis sydowiana* PPR extract on *Pseudomonas aeruginosa* PAO1; (B) Growth curve analysis of *P. aeruginosa* PAO1 treated with 250 and 500 μg/ml concentrations of fungal crude extract; (C)Effect of crude extract on QS-regulated virulence factors of *Pseudomonas aeruginosa* PAO1; (D) Effect of sub-MIC concentration of crude extract on biofilm attributes of *P. aeruginosa* PAO1.

The MIC was calculated using concentrations of fungal extract from 3.90 to 4,000 μg/ml. The fungal extract did not exhibit bactericidal effect on *P. aeruginosa* (Fig. 2A). The MIC of the fungal extract against *P. aeruginosa* PAO1 was 1,000 μg/ml; hence, further experiments were performed at sub-MIC concentrations of 250 and 500 μg/ml. The effect of sub-MIC concentrations of fungal extract on the growth curves of *P. aeruginosa* PAO1 (Fig. 2B) revealed that neither of the sub-MIC concentrations (250 and 500 μg/ml) exhibited significant effect on the growth of the test pathogen; however, a reduction in the growth rate was detected at 500 μg/mL concentration, which could affect the bacterial QS.

### Effect of Fungal Extract on Violacein Production and Virulence Traits of *P. aeruginosa* PAO1

Violacein synthesis in *C. violaceum* was determined in the presence or absence of crude extract of PPR isolate and found to reduce with the increase in the concentration of fungal extract. The test results revealed inhibition in the violacein production by 80.86 ± 2.36% and 92.58 ± 4.73% at concentrations of 250 and 500 μg/ml of fungal extract, respectively.

The ability of fungal extract in reducing the QS-dependent virulence phenotypes of *P. aeruginosa* PAO1 is illustrated in Fig. 2C. The activity of virulence phenotypes such as pyocyanin, protease, elastase, chitinase, and staphylolytic activity of *P. aeruginosa* PAO1 was downregulated by fungal extract in a dose-dependent manner. The results of pyocyanin production revealed 54.22 ± 6.41% and 64.38 ± 4.91% reduction in the test bacterium on treatment with fungal extract (250 and 500 μg/ml), respectively. The activities of different lytic enzymes including protease (68.05 ± 5.93%), elastase (62.61 ± 4.23%), and chitinase (69.86 ± 5.91%) were also considerably reduced on treatment with a 500 μg/ml concentration fungal extract at sub-MIC concentrations (Fig. 2C). The staphylolytic activity was inhibited by 62.09 ± 2.48% and 73.70 ± 3.16% on treatment with 250 and 500 μg/ml concentrations of fungal extract, respectively.

### Inhibition of Motility and Microscopic Observation of Biofilms

**Fig. 3:**
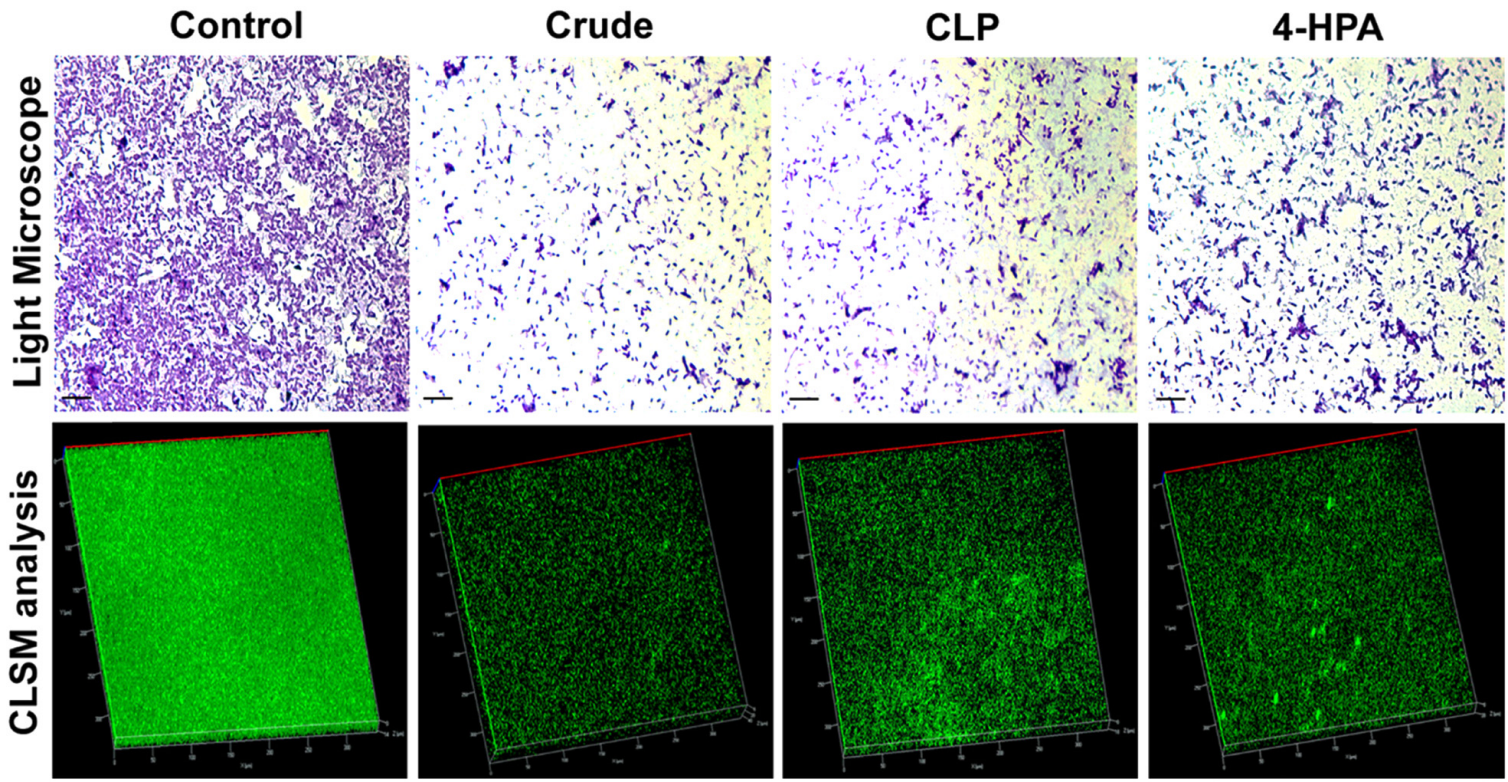
Microscopic observation of antibiofilm activity of fungal extract *Pestalotiopsis sydowiana* PPR and its bioactive compounds at sub-MIC concentration against *Pseudomonas aeruginosa* PAO1.

The effect of fungal extract in suppressing both swimming and swarming motility of *P. aeruginosa* PAO1 is illustrated in Fig. S1. The fungal extract at a concentration of 500 μg/ml exhibited comparatively higher inhibition rates of both swimming and swarming motility in *P. aeruginosa* PAO1. The observation of biofilm under light microscope and CLSM revealed the effect of fungal extract on biofilm, and the fungal extract (500 μg/ml)-treated samples presented scattered cells with less intensity as compared to the developed biofilm in the control (Fig. 3).

### Effect of Fungal Extract on Biofilm of *P. aeruginosa* PAO1

The crude extract of PPR exhibited significant inhibition of *P. aeruginosa* PAO1 biofilm development (Fig. 2D). The percentage of antibiofilm activity was observed as 54.30 ± 1.17% and 72.42 ± 3.89% at 250 and 500 μg/ml concentrations of the fungal extract.

### Inhibition of EPS, Rhamnolipid, and Alginate Production

The qualitative detection of the EPS production performed using Congo red plates is presented in Fig. S1. The control plate with black-colored colonies of *P. aeruginosa* PAO1 on Congo red agar indicated the production of the EPS, whereas the Congo red plate mixed with sub-MIC concentration (500 μg/ml) of fungal extract revealed the growth of test bacterium without black-colored colonies indicating the inhibition of EPS production. The production of EPS in *P. aeruginosa* PAO1 treated with 250 μg/ml concentration of fungal extract was 60.80 ± 6.69%; whereas, about 74.99 ± 2.90% of EPS inhibition was observed at 500 μg/ml concentration of fungal extract (Fig. 2D). Rhamnolipid production in *P. aeruginosa* is an important virulence factor that plays a pivotal role in cell adhesion and biofilm formation. The synthesis of rhamnolipid (43.14 ± 3.69% and 61.09 ± 5.16%) by *P. aeruginosa* PAO1 was inhibited on treatment with 250 and 500 μg/ml concentrations of fungal crude extract, respectively. These results suggest the effect of fungal extract on rhamnolipid production of *P. aeruginosa* PAO1 in a dose-dependent manner (Fig. 2D). Alginate is a major component of *P. aeruginosa* PAO1 during biofilm development. About 38.20 ± 3.64% and 60.37 ± 3.03% inhibition in production of alginate was observed at concentrations of 250 and 500 μg/ml of fungal crude extract treated *P. aeruginosa* PAO1, respectively (Fig. 2D).

The results of GC-MS analysis revealed the presence of different metabolites in the fungal extract (Table S2). The major bioactive compounds present in *P. sydowiana* PPR extract are 2-aminoacetophenone, 2-furoic acid, 2-phenylethanol, 2-(4-hydroxyphenyl) ethanol, N-phenethylacetamide, 4-HPA, and CLP.

### Molecular Modeling of RhlR and Molecular Docking

The 3D structure of the RhlR receptor molecule was modeled from the protein sequence retrieved from UniPort database (ID: P54292.1) using the ROBETTA on-line web server for structure modeling. The overall stereo chemistry quality assessment of the modeled RhlR structure was performed in the RAMPAGE web server. The overall stereochemistry qualities of built 3D-model structures of RhlR were accessed based on the psi/phi Ramachandran plot and the result demonstrated 100% presence of the amino acid residues in the favored region.

Molecular docking conferred better information about the interaction of bioactive compounds with LasR and RhlR receptor proteins. Natural ligands such as 3-oxo-C_12_-HSL and C_4_-HSL were used as reference models in molecular docking analyses for ligand interacting domains of LasR and RhlR, respectively. The molecular docking studies of different bioactive compounds identified in the crude extract of *P. sydowiana* PPR revealed strong interaction with the receptor proteins. CLP exhibited the highest docking score of −6.572 kcal/mol and it formed hydrogen bonds with residues Trp60, Thr115, and Ser129. These interactions and the score were comparatively equal to the natural ligand for LasR (Fig. S2). On the other hand, CLP had a docking score of -6.383 kcal/mol with RhlR receptor and formed hydrogen bond with residue TRP-68, which is relatively higher than their respective natural ligands and positive controls (Fig. S3). Thus, we concluded that the compound CLP exhibited higher affinity than other compounds toward the QS receptor proteins (Table S3).

### Molecular Dynamics Simulation

**Fig. 4:**
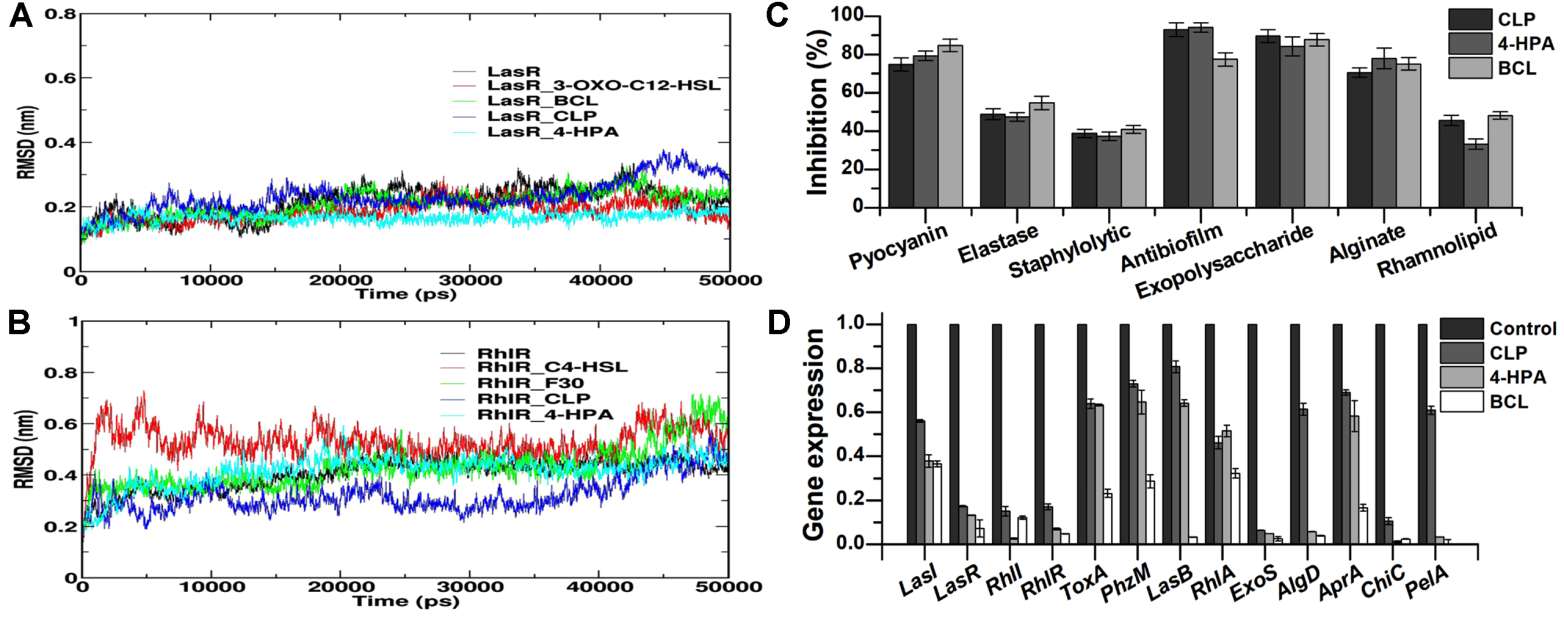
Root-mean-square deviation (RMSD) analysis with time for cyclo(-Leu-Pro) (CLP), 4-Hydroxyphenylacetamide (4-HPA), baicalein, and furanone C-30 along with (A) LasR and (B) RhlR. (**C**) Effect of bioactive compounds on the production of QS-regulated virulence factors, biofilm, and its determinants in *P. aeruginosa* PAO1. (**D**) Relative expression levels of QS-related genes of *P. aeruginosa* PAO1 exposed with bioactive compounds. Normalized with the reference gene *pr°C.* The error bar symbolized the standard deviation of the three independent values.

Molecular dynamics simulation studies were conducted to determine the conformational modification in LasR QS receptor protein in the presence of signaling molecules and bioactive compounds. The simulations were performed with four complexes, LasR-C_12_HSL, LasR-BCL, LasR-CLP, and LasR-4-HPA. Similarly, the complexes of these ligands with RhlR were also subjected to the simulation process. The simulations were run for 50,000 ps with the time step of 2 fs. The root-mean-square deviation (RMSD) profile was generated to analyze the interaction of QS receptor proteins with the signaling molecules and bioactive compounds throughout the simulation process. The RMSD values of the complexes formed by QS receptors with their respective natural ligands and bioactive compounds were within the range of 2Å, indicating accurate docking [45]. Overall, the complexes were highly stable and RMSD values were maintained below 0.4 nm. Individual analysis based on RMSD showed that the LasR-3-oxo-C_12_HSL, LasR-BCL, LasR-CLP, and LasR-4HPA complexes were stable after ~12,000 ps, ~14,000 ps, ~20,000 ps, and ~15,000 ps, respectively. The fluctuations observed after ~35,000 ps and ~10,000 ps as well as the stability were maintained till the end of the simulation (Fig. 4A). In the case of RhlR, RMSD values of all the complexes were maintained below 0.8 nm. Individual analysis showed that the complex RhlR-C_4_-HSL was stable between ~10,000 ps and 40,000 ps, whereas RhlR-F30 attained stability at ~20,000 ps, and fluctuation was observed after 40,000 ps. RhlR-CLP maintained its stability between ~10,000 ps and 40,000 ps, whereas RhlR-4HPA complex was highly stable from ~10,000 ps and maintained its stability till the end of the simulation (Fig. 4B). The binding energy for LasR complex formation was −122.316 kJ/mol for LasR-C_12_-HSL, −164.58 kJ/mol for LasR-Baicalein, −52.930 kJ/mol for LasR-CLP and −100.375 kJ/mol for LasR-4HPA. In case of RhlR complexes, the binding energy values were −51.274 kJ/mol for RhlR-C_4_HSL, −93.732 kJ/mol for RhlR-Furanone C-30, −35.813 kJ/mol for RhlR-CLP and −95.881 kJ/mol for RhlR-4HPA (Table 2).

### Effect of Bioactive Compounds of *P. sydowiana* PPR on the Growth of *P. aeruginosa* PAO1

The MIC of CLP, 4-HPA, and BCL (positive control) against *P. aeruginosa* PAO1 were determined. The MIC values of CPL and BCL against *P. aeruginosa* PAO1 were found to be 250 μg/ml, whereas the MIC of 4-HPA from *P. sydowiana* PPR was 125 μg/ml. Hence, the in vitro anti-QS and anti-biofilm activities of the selected bioactive compounds (CLP and BCL) were evaluated at the sub-MIC concentration of 125 μg/ml. The in vitro anti-QS and anti-biofilm activities of 4-HPA were determined at the sub-MIC concentration of 62.5 μg/ml. The sub-MIC concentrations of each bioactive compound and positive control were investigated for their effect on the growth of *P. aeruginosa* PAO1 using growth curve analysis. The experimental results revealed that the sub-MIC concentration had no adverse effect on the growth of *P. aeruginosa* PAO1 and growth curve patterns were similar to those of untreated control.

### Anti-QS Potential of Bioactive Compounds

Pyocyanin is an important virulence factor secreted by *P. aeruginosa*, which is regulated by genes controlled by QS. The effect of fungal bioactive compounds on the synthesis of pyocyanin was evaluated. The results showed that the bioactive compounds from *P. sydowiana* PPR significantly affected the synthesis of pyocyanin by *P. aeruginosa* PAO1. At the sub-MIC concentration, CLP, 4-HPA, and BCL significantly inhibited the pyocyanin synthesis by *P. aeruginosa* PAO1 with inhibition of 74.72 ± 3.42%, 79.22 ± 2.41% and 84.73 ± 3.28%, respectively. CLP, 4-HPA, and BCL significantly inhibited elastase synthesis by *P. aeruginosa* with inhibition of 48.78 ± 2.82%, 47.31 ± 2.27% and 54.76 ± 3.49%, respectively. The fungal bioactive compounds were evaluated for their ability to inhibit the staphylolytic activity of *P. aeruginosa* PAO1. At the sub-MIC concentration, CLP, 4-HPA, and BCL significantly inhibited the staphylolytic activity of *P. aeruginosa* PAO1 with inhibition of 38.71 ± 2.11%, 37.25 ± 2.29% and 40.83 ± 1.97%, respectively (Fig. 4C). In addition to the QS-regulated virulence factors, the selected bioactive compounds significantly inhibited the biofilm formation too. At the sub-MIC concentration, CLP, 4-HPA, and BCL significantly inhibited the biofilm formation by *P. aeruginosa* PAO1 with inhibition of 92.91 ± 3.56%, 94.10 ± 2.51% and 77.37 ± 3.51%, respectively. On treatment with sub-MIC concentrations of *P. sydowiana* PPR bioactive compounds, the synthesis of QS-regulated biofilm determinants such as exopolysaccharides (EPS), alginates, and rhamnolipids was also significantly inhibited (Fig. 4C). Moreover, a significant reduction was observed in the swimming and swarming motility of *P. aeruginosa* PAO1 when treated with sub-MIC concentrations of CLP, 4-HPA, and BCL.

### Gene Expression Studies

The RT-PCR results revealed the mRNA expression levels of different QS-regulated virulence genes of *P. aeruginosa* PAO1 on treatment with bioactive compounds CLP, 4-HPA, and BCL (positive controls). The expression levels of selected genes including *LasI, LasR, RhlI, RhlR, ToxA, PhzM, LasB, RhlA, Exo, AlgD, AprA, ChiC*, and *PelA* were significantly reduced when treated with sub-MIC dosages in comparison to the untreated controls (Fig. 4D).

## Discussion

QS is a regulatory process that allows a bacterial population to collectively express various virulence factors associated with pathogenesis including biofilm formation [47-50]. Targeting the QS circuits of bacteria has been found to be a promising strategy to combat bacterial infections as an alternative to conventional antibiotics [51]. This therapeutic strategy potentially inhibits the production of pathogenic phenotypes of the bacteria without provoking any adverse effect on their growth. The marine ecosystem appeared to be a promising source of diverse biological active compounds with pharmaceutical applications [52]. The metabolites of different marine-derived bacteria, actinomycetes, and fungi were reported as potential inhibitors of QS and its regulatory factors [53]. In the present study we examined the potential of the marine-derived fungi as a source of antipathogenic molecules.

Zhang *et al.* [54] reported the anti-QS and antibiofilm activities of equisetin isolated from a marine fungus *Fusarium* sp. Z10 against *P. aeruginosa* PAO1. Screening of 14 fungal isolates as QSIs revealed that PPR exhibited significant effect. The fungal species *Pestalotiopsis* is known to have antimicrobial potential but not as QSI against *P. aeruginosa.* [55]. The present results revealed that the fungal extract significantly inhibited the *las* system, which was related to decrease in the production of elastase, alkaline protease, pyocyanin, chitinase, rhamnolipid, and swarming motility. This fungal isolate could significantly attenuate the QS-governed virulence traits in *P. aeruginosa* PAO1.

The present study also presented the effect of fungal extract on the production of rhamnolipids, EPS, and alginate, the major components of the biofilm [30, 56]. Molecular docking study revealed that the metabolite (cyclo(-Leu-Pro) of PPR isolate adapts in the structure of receptor protein in a likely fashion to the natural ligands and positive controls (Figs. S2 and S3). RMSD profile revealed that throughout the simulation, complexes of both the QS receptors with bioactive compounds were equally stable when compared with the LasR signaling molecule complex. Hnamte *et al.* [46] suggested that docking analysis may be useful to demonstrate the anti-QS efficiency of phytochemicals. Molecular dynamics simulation studies demonstrated that mosloflavone can act as an anti-QS agent against *P. aeruginosa* PAO1.

Among the different metabolites of *P. sydowiana* PPR, two bioactive compounds, CLP and 4-HPA, were determined as significant compounds and shown to inhibit the synthesis of QS-regulated virulence determinants and biofilm formation in *P. aeruginosa* PAO1. To the best of our knowledge, no attempt has been made to explore the anti-QS and antibiofilm activities of these bioactive compounds from *P. sydowiana* PPR against *P. aeruginosa* PAO1. The experimental results of this study showed the potential of these bioactive compounds to inhibit the synthesis of QS-regulated pathogenic factors and biofilm formation. These compounds can be used as anti-QS agents to exert non-lethal effect on the growth of *P. aeruginosa* PAO1, to abate the possibility of drug resistance.

As mentioned previously, QS plays a significant role in the pathogenicity of *P. aeruginosa* PAO1 by regulating various virulence genes and subsequently mediating the synthesis of virulence factors. The major QS-related genes as well as virulence genes, which are regulated by the QS system in *P. aeruginosa* PAO1, were analyzed using RT-PCR to evaluate the expression level when the bacterial strain was treated with or without the bioactive compounds. The experimental results of gene expression analysis revealed that the bioactive compounds significantly downregulated the expression of all the genes analyzed. In particular, the expression levels of *lasI, lasR, rhlI*, and *rhlR* were significantly reduced (Fig. 4D). Since *lasI/R* and *rhlI/R* are the major regulatory genes of the *P. aeruginosa* PAO1 QS system, targeted downregulation of these genes would inhibit the QS circuit, which can subsequently impair the pathogenicity associated with the QS system.

In conclusion, the effect of metabolites from *P. sydowiana* PPR on QS system and its regulatory pathogenic phenotypes in *P. aeruginosa* PAO1 were revealed. The bioactive compounds produced by the fungus exhibited promising inhibition of virulence traits, motility, and biofilm formation. The molecular docking and molecular dynamics simulation studies revealed a significant interaction of fungal metabolites with the QS receptor proteins of test bacterium. The present study demonstrated for the first time that CLP and 4-HPA produced by *P. sydowiana* PPR suppress the synthesis of several QS-associated virulence factors in *P. aeruginosa* PAO1 without affecting the bacterial growth. These results concluded that the metabolites of *P. sydowiana* PPR could potentially inhibit the QS system and limit the degree of pathogenicity in *P. aeruginosa* PAO1.

## Supplementary material

Supplementary data for this paper are available on-line only at http://jmb.or.kr.



## Figures and Tables

**Table 1:** Preliminary screening of extract of different fungal isolates against QS systems in the biosensor strain and test pathogen.

Sample Code	Zone of inhibition (mm)			

	*Chromobacterium violaceum*		*Pseudomonas aeruginosa* PAO1	

	250 μg/ml	500 μg/ml	250 μg/ml	500 μg/ml
PPR	19.33 ± 1.52	24.67 ± 1.15	14.33 ± 2.08	19.00 ± 1.00
MC1	16.30 ± 1.53	16.30 ± 1.53	11.70 ± 1.53	14.00 ± 2.00
PM6	12.33 ± 0.57	14.00 ± 2.64	11.67 ± 1.52	15.33 ± 1.15
De20	11.67 ± 0.57	12.67 ± 1.52	9.00 ± 0.57	12.67 ± 0.57
DM19	11.00 ± 1.00	14.67 ± 1.52	8.33 ± 0.57	12.00 ± 1.00
DM15	10.67 ± 0.57	9.33 ± 1.52	9.66 ± 1.52	10.33 ± 0.57
DM2	11.00 ± 1.00	12.33 ± 2.08	8.00 ± 0.00	10.33 ± 1.52
DM38	11.00 ± 1.00	9.33 ± 1.52	8.00 ± 0.00	8.66 ± 1.15
DE29	9.66 ± 1.52	13.33 ± 1.15	10.00 ± 1.00	12.00 ± 2.00
DM32a	9.00 ± 1.00	10.33 ± 1.15	8.33 ± 0.57	8.33 ± 0.57
DM25	8.66 ± 0.57	13.00 ± 1.00	9.00 ± 1.00	11.00 ± 1.00
DE27	8.33 ± 0.57	13.33 ± 1.15	8.66 ± 1.15	9.33 ± 1.15
DE09	8.33 ± 0.57	11.33 ± 1.52	8.00 ± 0.00	12.00 ± 0.00
DM33	8.33 ± 0.57	11.67 ± 1.52	8.00 ± 0.00	9.00 ± 1.00

**Table 2:** MM/PBSA analysis: LasR and RhlR QS circuit with binding energy and their constituents (kJ/mol).

Ligand ID	Van der Waals energy (kJ/mol)	Binding energy (kJ/mol)	Electrostatic energy (kJ/mol)	Polar salvation energy (kJ/mol)	SASA energy (kJ/mol)
LasR	-171.224	-122.316	-53.143	119.778	-17.700
C_12_-HSL					
CLP	-119.284	-52.930	-34.116	112.670	-12.179
4-HPA	-157.057	-100.375	-122.486	193.563	-14.433
BCL	-190.142	-164.580	-3.662	45.533	-16.303
RhlR	-105.598	-51.274	-45.407	111.066	-11.321
C_4_-HSL					
Furanone C30	-122.381	-93.732	-12.116	50.993	-10.194
CLP	-78.249	-35.813	-22.521	73.401	-8.499
4-HPA	-109.390	-95.881	-63.915	88.025	-10.654
