# Corrigendum to: Inhibition of Microbial Quorum Sensing Mediated Virulence Factors by *Pestalotiopsis sydowiana*

**DOI:** 10.4014/jmb.2020.3007.1108

**Published:** 2020-07-28

**Authors:** Paramanantham Parasuraman, B Devadatha, V. Venkateswara Sarma, Sampathkumar Ranganathan, Dinakara Rao Ampasala, Dhanasekhar Reddy, Ranjith N. Kumavath, Sanjay K. S. Patel, Vipin Chandra Kalia, Jung-Kul Lee, Busi Siddhardha

**Affiliations:** 1 Department of Microbiology, School of Life Sciences, Pondicherry University, Puducherry 605014, India; 2 Department of Biotechnology, School of Life Sciences, Pondicherry University, Puducherry 605014, India; 3 Centre for Bioinformatics, School of Life Sciences, Pondicherry University, Puducherry 605014, India; 4 Department of Chemical Engineering, Konkuk University, Hwayang-Dong, Gwangjin-Gu, Seoul 05029, Republic of Korea; 5 Department of Genomic Science, School of Biological Sciences, Central University of Kerala, Tejaswini Hills, Periya (P.O), Kasaragod, Kerala 671320, India

In the article titled “**Inhibition of Microbial Quorum Sensing Mediated Virulence Factors by *Pestalotiopsis sydowiana***”, the authors noticed that the Hydrogen bond between ligands and LasR and RhlR receptor proteins were not mentioned properly in the Fig. S2 and S3. The corrected Fig. S2 and Fig. S3 are now available online.

The authors sincerely apologize for the confusion caused by this error and state that this does not change the scientific conclusions of the article in any way.



