# Supplementary material for: Inhibition of Microbial Quorum Sensing Mediated Virulence Factors by Pestalotiopsis sydowiana
Source: J Microbiol Biotechnol. 2020 Jan 23;30(4):571–82. doi: 10.4014/jmb.1907.07030 (PMC9728384; doi:10.4014/jmb.1907.07030)
Supplement: Supplementary file 1 [file JMB-30-4-571-supple.pdf]

J. Microbiol. Biotechnol.
https://doi.org/10.4014/jmb.1907.07030

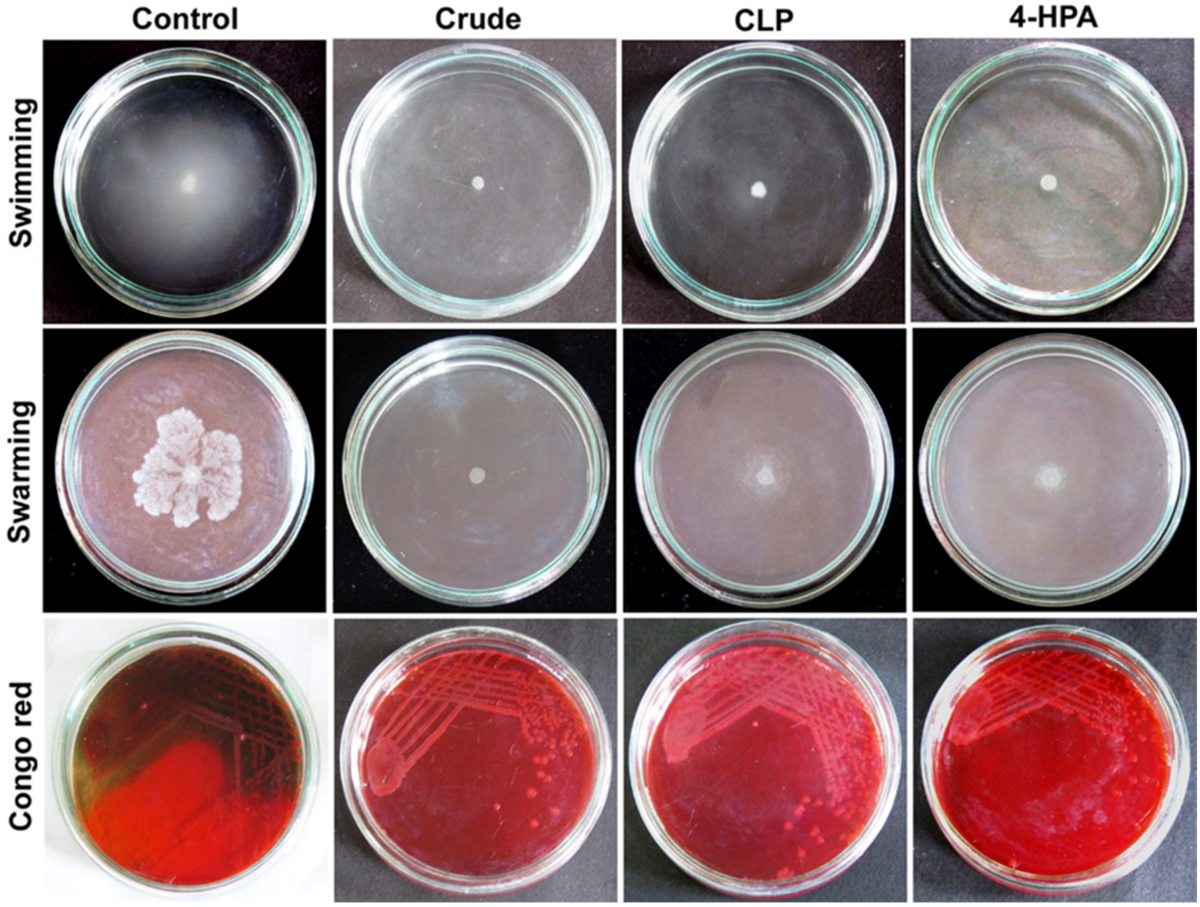

Fig. S1. Inhibition of swimming and swarming motility of *Pseudomonas aeruginosa* PAO1 by fungal extract of *Pestalotiopsis sydowiana* PPR and their bioactive compounds. Congo red agar plates presenting the effect of the crude extract and the bioactive compounds on the EPS production of *P. aeruginosa* PAO1.

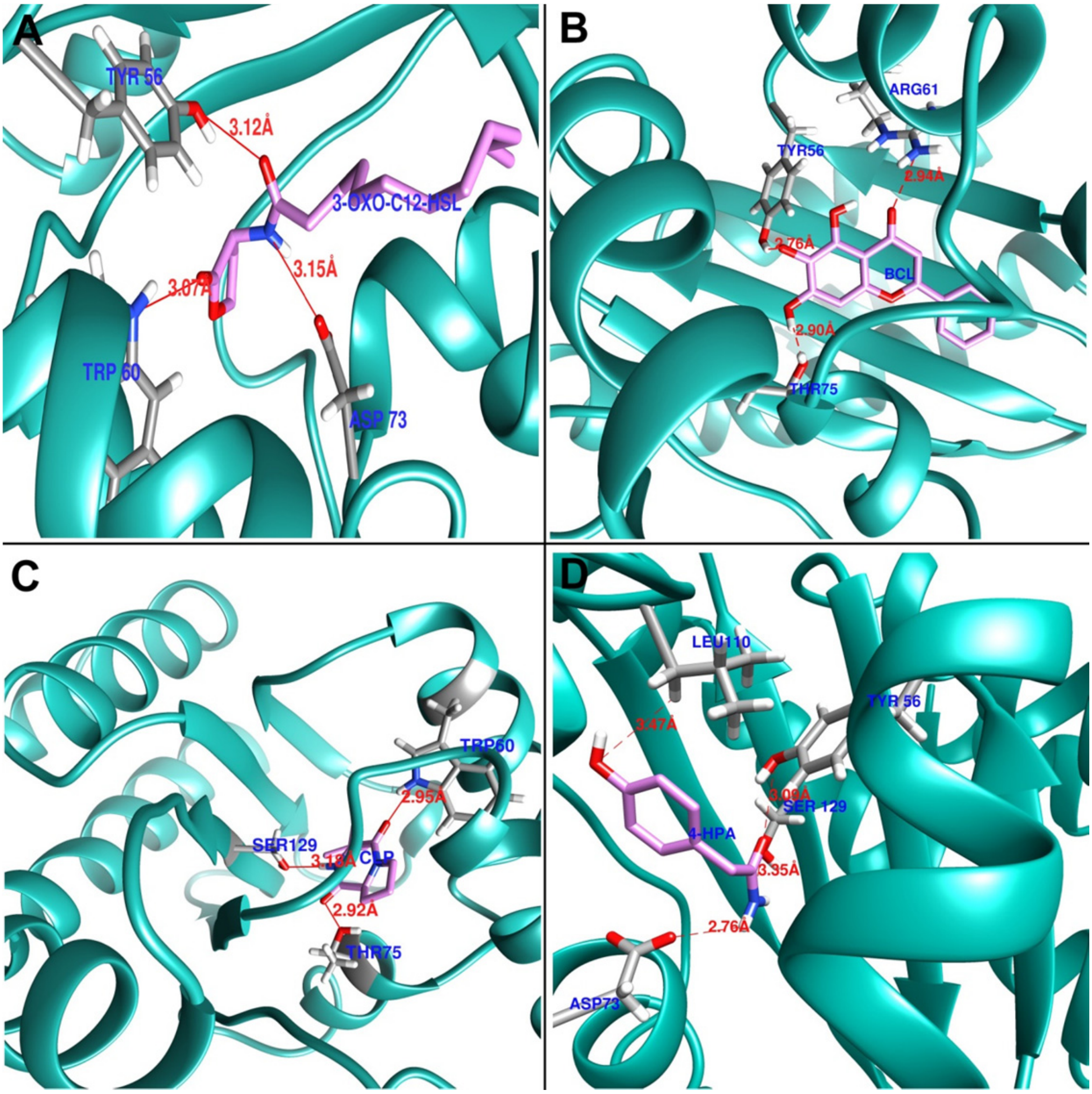

Fig. S2. Molecular docking conformation of natural ligand (3-oxo-C_{12}-HSL), positive control (baicalein), and fungal metabolites from *Pestalotiopsis sydowiana* PPR with active sites of the QS receptor protein, LasR. (A) 3-oxo-C_{12}-HSL, (B) baicalein (BCL), (C) cyclo (-Leu-Pro) (CLP), (D) 4-Hydroxyphenylacetamide (4-HPA).

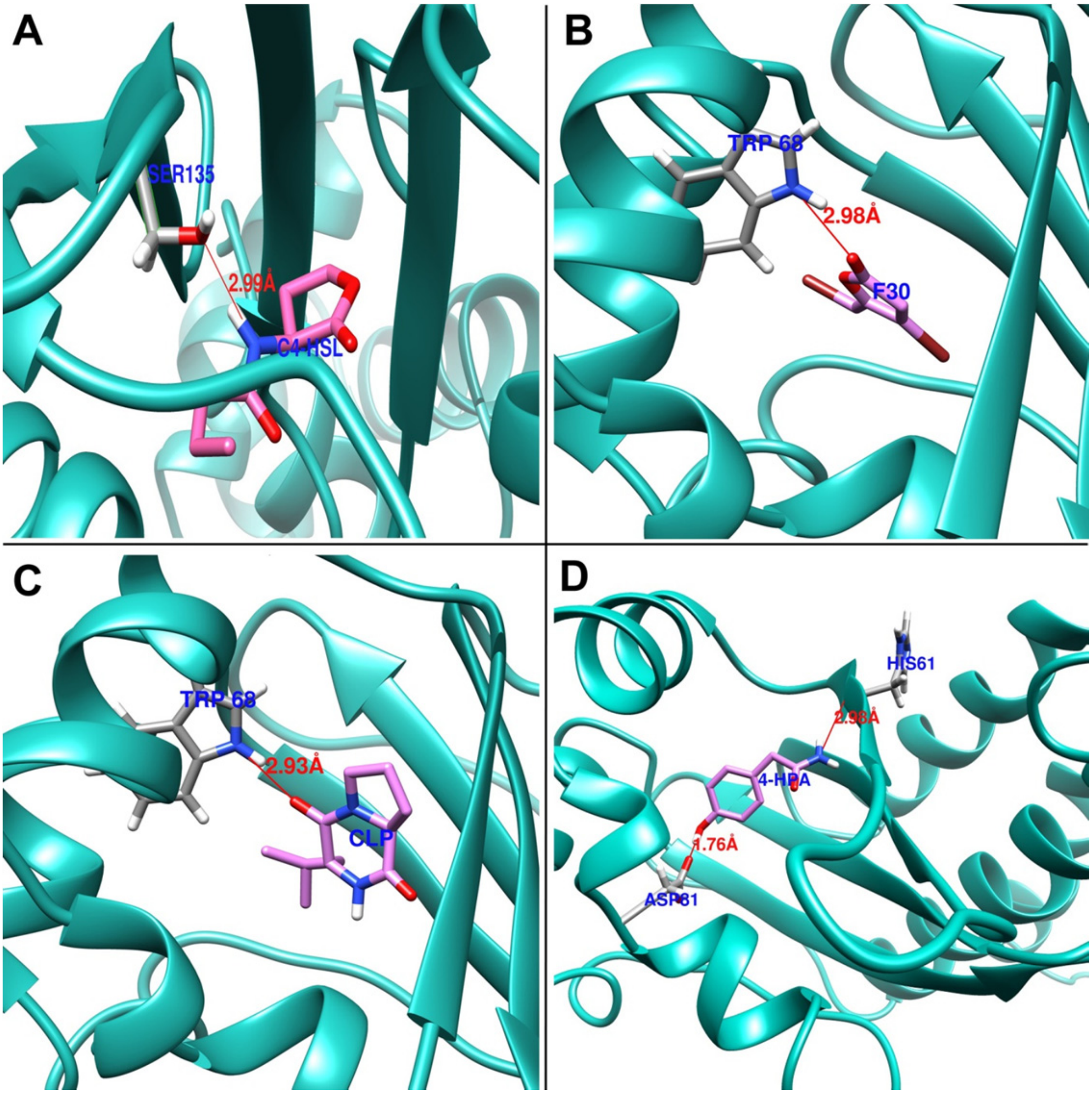

Fig. S3. Molecular docking conformation of natural ligand (C_4-HSL), positive control, (furanone C30) and fungal metabolites from *Pestalotiopsis sydowiana* PPR with active sites of the QS receptor protein, RhlR. (A) C_4-HSL, (B) furanone C30 (F30), (C) cyclo(-Leu-Pro) (CLP), (D) 4-Hydroxyphenylacetamide (4-HPA).

Table S1. List of primers for *P. aeruginosa* QS genes for RT-PCR.

Gene	Primer	Sequence (5'-3')	Melting temp.	Amplicon size (bp)	Reference
lasI	Forward	GGCTGGGACGTTAGTGTCAT	59°C	104	[1]
	Reverse	AAAACCTGGGCTTCAGGAGT			
lasR	Forward	ACGCTCAAGTGGAAAATTGG	56°C	111	
	Reverse	TCGTAGTCCTGGCTGTCCTT			
rhlI	Forward	AAGGACGTCTTCGCCTACCT	59°C	130	
	Reverse	GCAGGCTGGACCAGAATATC			
rhlR	Forward	CATCCGATGCTGATGTCCAACC	51°C	101	
	Reverse	ATGATGGCGATTTCCCCGGAAC			
phzM	Forward	GAATGGAAGTCCCGTTGC	53°C	225	
	Reverse	GCCCTCGACATCCCTCA			
lasB	Forward	GACCGAGAATGACAAAGTGGAA	51°C	80	
	Reverse	GGTAGGAGACGTTGTAGACCAGTTG			
rhlA	Forward	TGGCCGAACATTTCAACGT	51°C	107	
	Reverse	GATTTCCACCTCGTCGTCCTT			
toxA	Forward	GGAGCGCAACTATCCCACT	53°C	150	
	Reverse	TGGTAGCCGACGAACACATA			
exoS	Forward	GGCGGATGCGGAAAAGTAC	55°C	`121	
	Reverse	CTGACGCAGAGCGCGATT			
aprA	Forward	CCCTGTCCTATTCGTTCCTG	53°C	175	
	Reverse	GCGTCGACGAAGTGGATATT			
algD	Forward	GCGACCTGGACCTGGGCT	55°C	145	
	Reverse	TCCTCGATCAGCGGGATC			
chiC	Forward	CTGGGAGTTCCGCAAGCGTTAC	59°C	268	
	Reverse	ATCGGTGGCGGTGACGAAATAG			
pelA	Forward	CCTTCAGCCATCCGTTCTTCT	55°C	118	
	Reverse	TCGCGTACGAAGTCGACCTT			
proC	Forward	CAGGCCGGGCAGTTGCTGTC	-	180	
	Reverse	GGTCAGGCGCGAGGCTGTCT			

Table S2. GC-MS literature of fungal metabolites in the crude extract of the *Pestalotiopsis sydowiana* PPR.

Fungal metabolites	Retention time (min)	Molecular formula	Molecular weight (g/mol)	Peak area (%)
2-Aminoacetophenone	3.092	C_8H_9NO	135.166	8.13
2-Furoic acid	7.708	$C_5H_4O_3$	112.084	0.64
2-Phenylethanol	9.050	$C_8H_{10}O$	122.167	5.95
2-(4-Hydroxyphenyl)ethanol	17.20	$C_8H_{10}O_2$	138.166	2.40
N-Phenethylacetamide	19.27	$C_{10}H_{13}NO$	163.220	3.12
4-hydroxyphenylacetamide	20.20	C_8H_9NO	151.165	1.68
Cyclo(-Leu-Pro)	28.53	$C_{11}H_{18}N_2O_2$	210.277	3.64

Table S3. Molecular docking analysis of fungal metabolites with QS receptors of *Pseudomonas aeruginosa* (LasR and RhlR).

Compound Name	LasR				RhlR			
	Docking Score (Kcal/mol)	Glide Emodel Score	Hydrogen Bonding	Hydrophobic Interaction	Docking Score (Kcal/mol)	Glide Emodel Score	Hydrogen Bonding	Hydrophobic Interaction
N-(3-oxododecanoyl)-L-homoserine lactone* (LasR)<br>N-Butyryl-L-homoserine lactone* (RhlR)	-6.928	-73.83	TYR56, TRP60, ASP73	TYR64, ALA105, PHE101, PRO74, TYR93, TRP88, LEU40, CYS79, LEU125, VAL76, ALA127, LEU39, ALA70, TYR47, ALA50, ILE52, LEU36, LEU110	-5.491	-38.87	SER135	ALA83, PHE101, TRP96, TRP68, ALA111, LEU107, TRP108, TYR72, LEU116, LEU69, TYR64, VAL60, TYR45, ALA44, ILE84
Baicalein** (LasR)<br>Furanone C30** (RhlR)	-5.495	-62.95	TYR56, THR75, ARG61	TYR64, LEU36, ILE52, ALA50, LEU39, LEU125, LEU40, CYS79, VAL76, ALA127, TRP88, LEU110, PHE101, TRP60	-4.772	-26.86	TRP68	ILE84, ALA83, TRP96, TRP108, LEU116, PHE101, ALA111, LEU107, TYR72, TYR64
Cyclo(-Leu-Pro)	-6.572	-28.87	TRP60, THR75, SER129	TYR64, ALA105, LEU110, PHE101, TYR93, TRP88, ALA127, VAL76, LEU36, TYR56	-6.383	-15.69	TRP68	ILE84, PHE101, ALA83, LEU116, TRP96, LEU107, TYR72, TYR64, LEU69, VAL60,

								ALA44, TYR45
4-Hydroxy phenylacetamide	-5.925	-39.38	TYR56, SER129, LEU110, ASP73	TYR64, ALA105, TRP60, TRP88, TYR93, ALA127, VAL76, LEU36	-6.192	-29.25	ASP81, HIS61	VAL60, ALA44, TYR45, ILE84, TRP96, LEU107, TRP68, LEU69
2-Aminoacetophenone	-5.644	-37.65	TRP60	TYR64, LEU36, VAL76, TRP88, LEU110, TYR93, ALA105, PHE101, TYR56	-5.684	-31.224	TRP68	LEU69, TYR45, ALA44, VAL60, VAL133, TRP96, ALA83, ILE84, TYR72, TYR64
2-(4-Hydroxyphenyl) ethanol	-5.421	-35.573	ASP73, SER129	TRP88, TYR93, ALA105, TRP60, TYR64, TYR56, LEU36, VAL76	-4.834	-29.13	ASP81	ALA44, ILE84, ALA83, PHE101, LEU107, TRP96, TYR72, TRP68, LEU69, TYR45, VAL60
2-Furoic acid	-5.114	-29.66	THR75, SER129, TYR56	LEU36, LEU110, TRP60, PHE101, ALA105, TYR93	-3.996	-20.69	TRP68	TYR72, TYR64, ILE84, ALA83, PHE101, LEU116, LEU107, TRP108, ALA111
N-Phenethylacetamide	-4.876	-33.29	SER129	TYR56, TYR64, LEU36, TRP60, LEU110, TYR93, ALA105, PHE101, TRP88, VAL76	-4.432	-25.53	TRP68	TYR72, VAL60, ALA44, ILE84, TRP96, TRP108, PHE101, ALA83, LEU107, LEU69

Reference

1. Hnamte S, Parasuraman P, Ranganathan S, Ampasala DR, Reddy D, Kumavath RN, Suchiang K, Mohanty SK, Busi S. 2019. Mosloflavone attenuates the quorum sensing controlled virulence phenotypes and biofilm formation in *Pseudomonas aeruginosa* PAO1: *In vitro*, *in vivo* and *in silico* approach. *Microb. Pathog.* **131:** 128-134.
